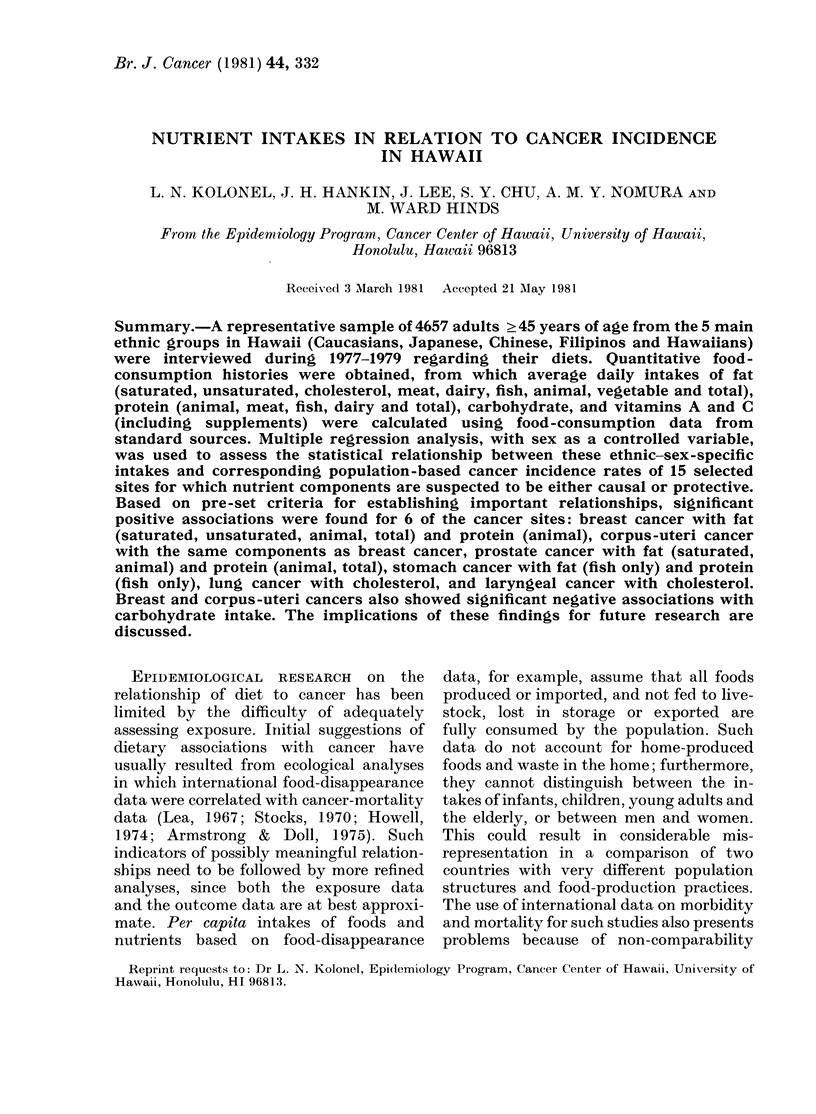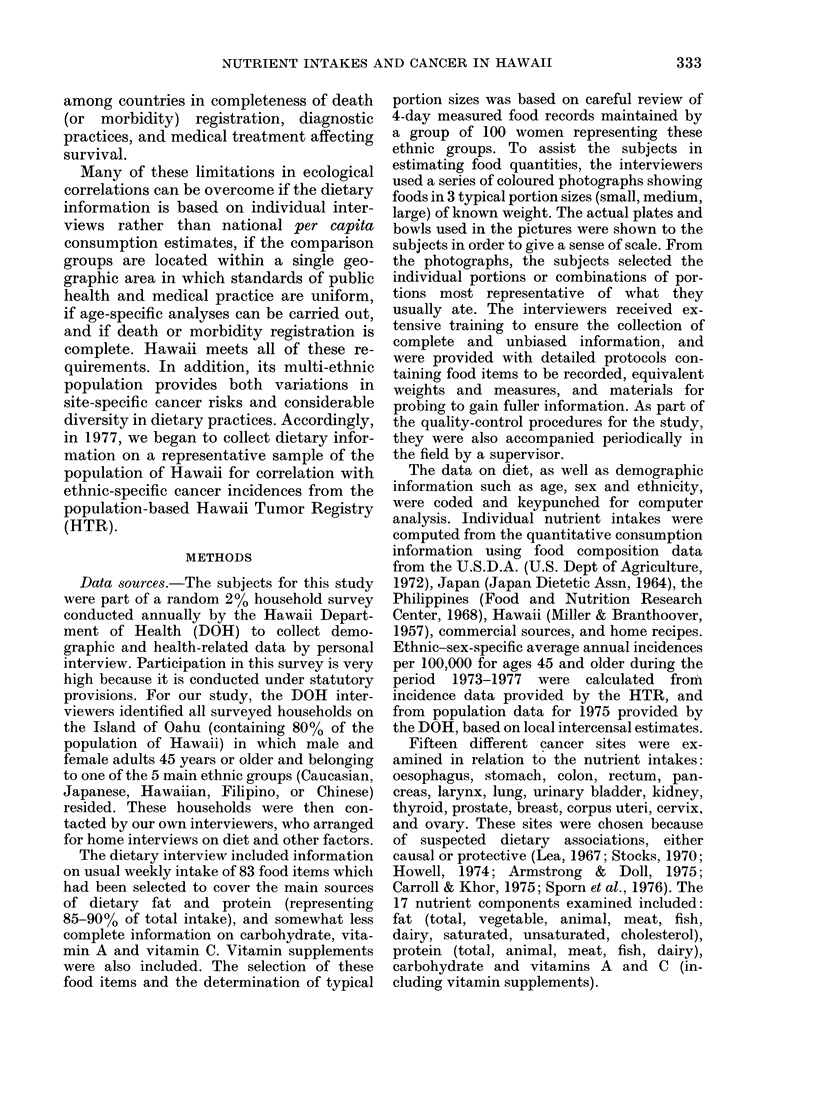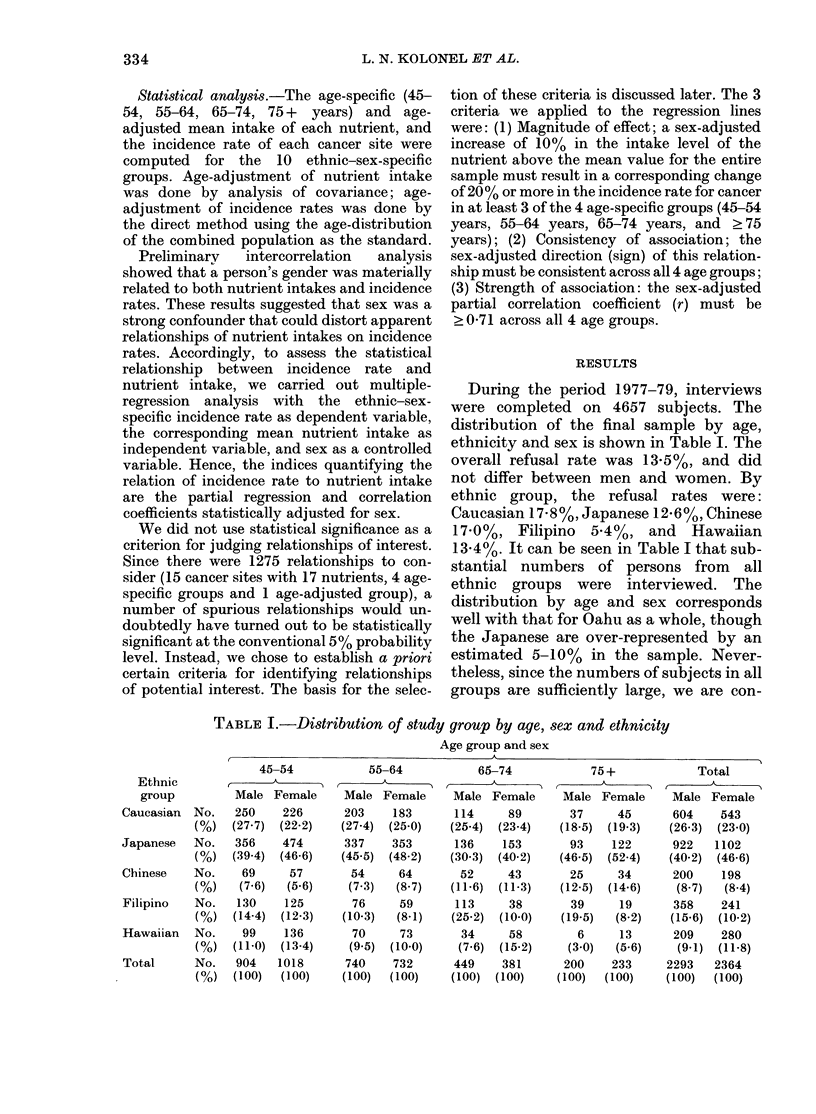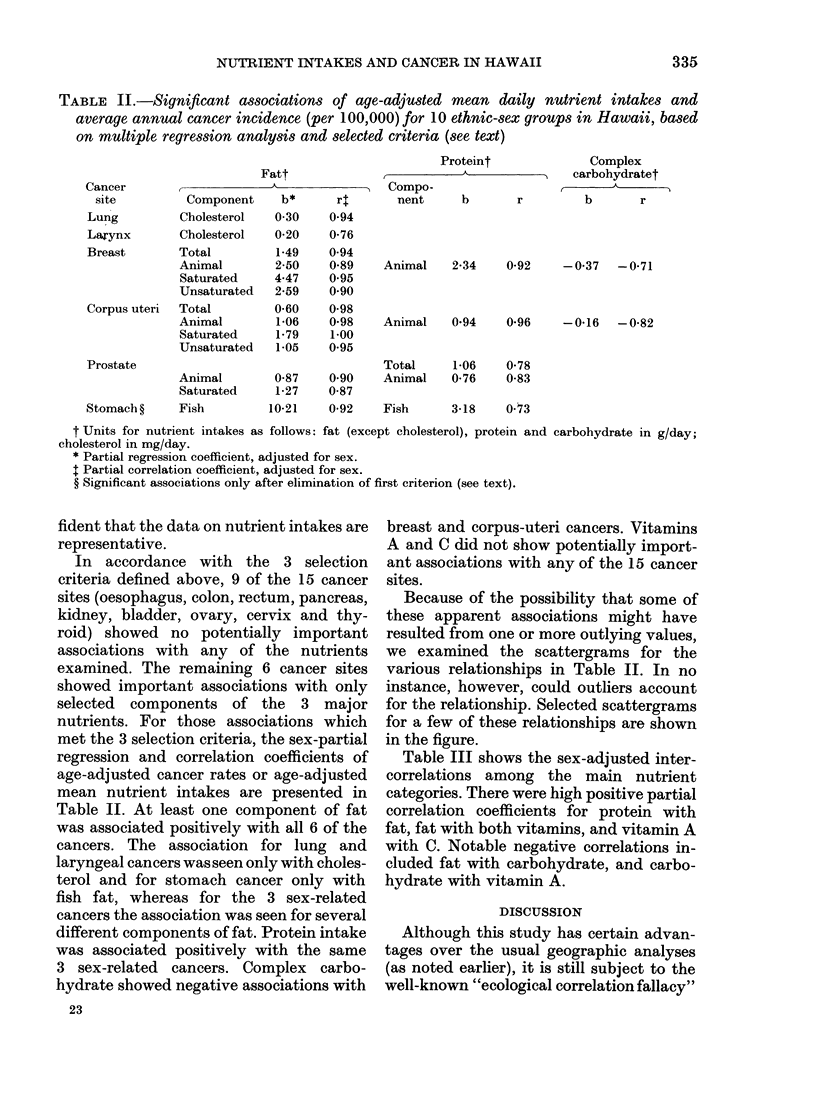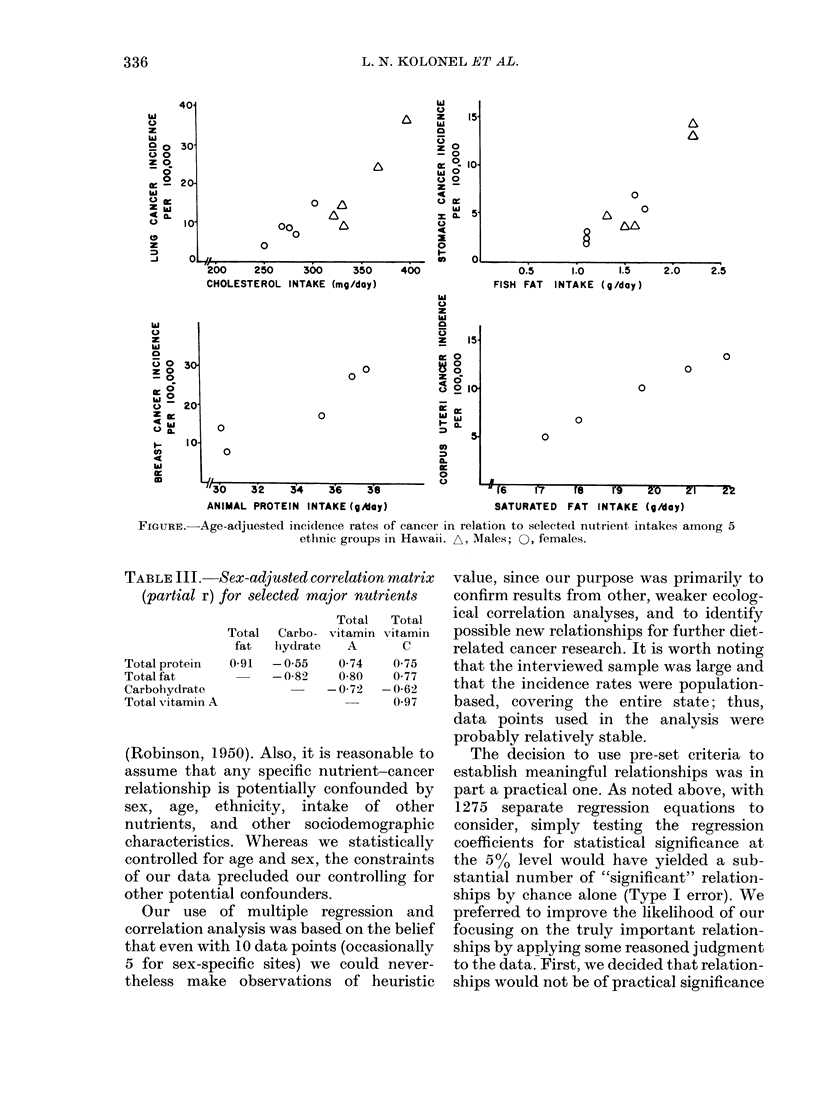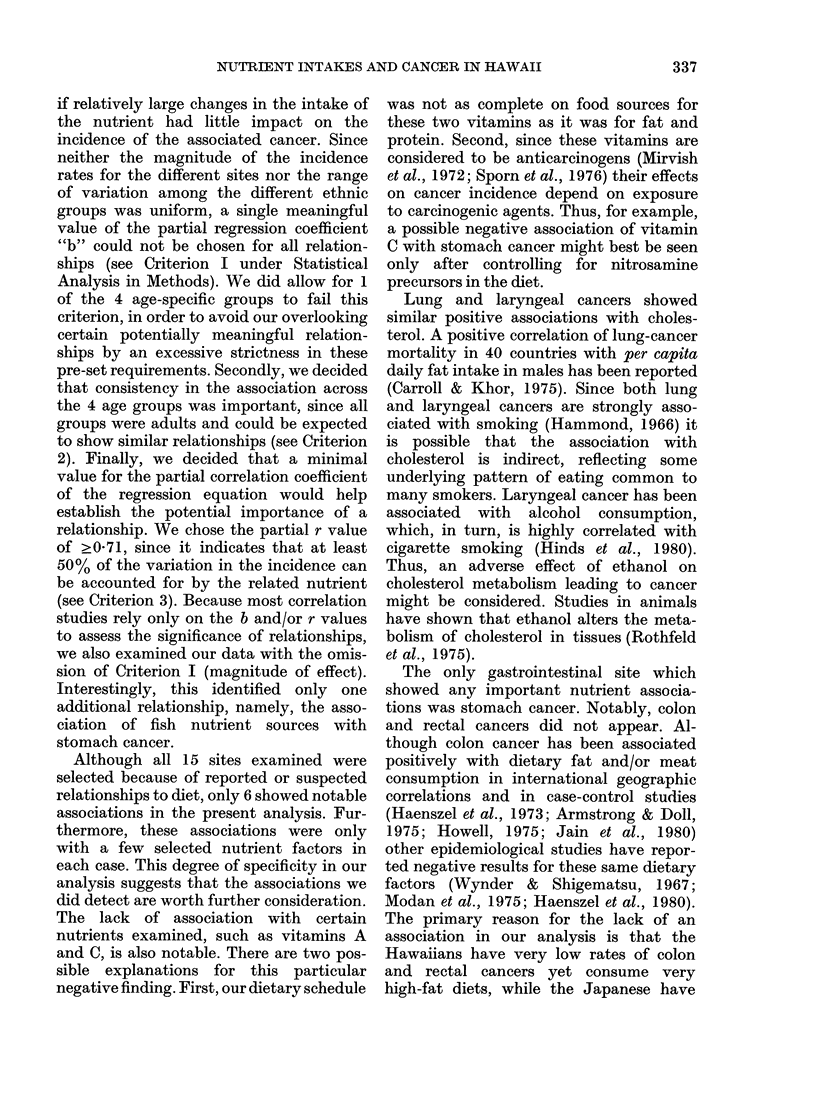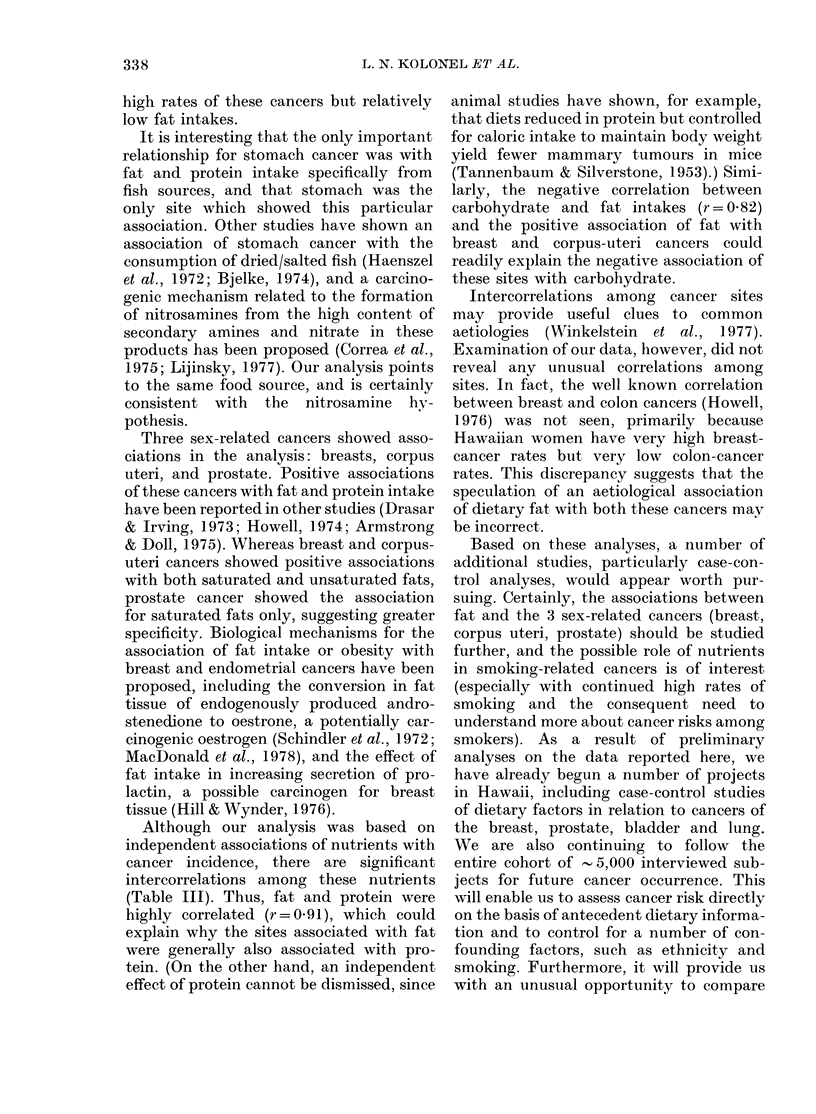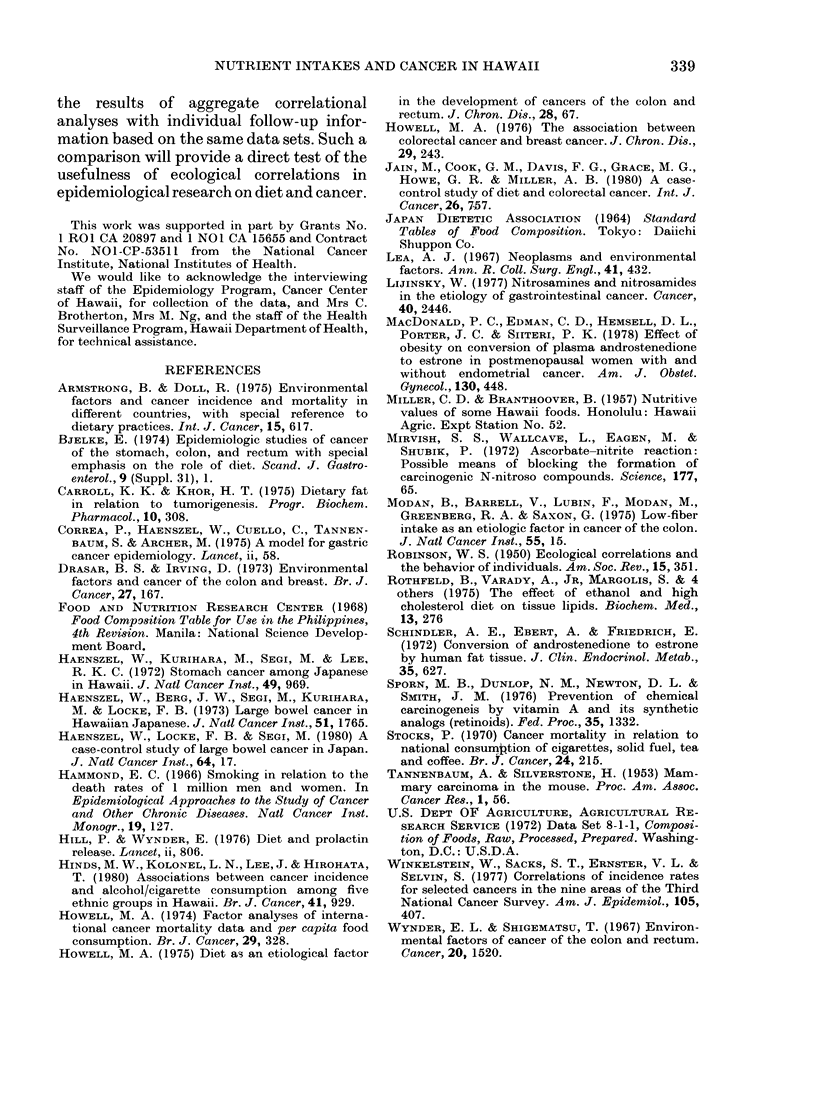# Nutrient intakes in relation to cancer incidence in Hawaii.

**DOI:** 10.1038/bjc.1981.189

**Published:** 1981-09

**Authors:** L. N. Kolonel, J. H. Hankin, J. Lee, S. Y. Chu, A. M. Nomura, M. W. Hinds

## Abstract

A representative sample of 4657 adults greater than or equal to 45 years of age from the 5 main ethnic groups in Hawaii (Caucasians, Japanese, Chinese, Filipinos and Hawaiians) were interviewed during 1977-1979 regarding their diets. Quantitative food-consumption histories were obtained, from which average daily intakes of fat (saturated, unsaturated, cholesterol, meat, dairy, fish, animal, vegetable and total), protein (animal, meat, fish, dairy and total), carbohydrate, and vitamins A and C (including supplements) were calculated using food-consumption data from standard sources. Multiple regression analysis, with sex as a controlled variable, was used to assess the statistical relationship between these ethnic-sex-specific intakes and corresponding population-based cancer incidence rates of 15 selected sites for which nutrient components are suspected to be either causal or protective. Based on pre-set criteria for establishing important relationships, significant positive associations were found for 6 of the cancer sites: breast cancer with fat (saturated, unsaturated, animal, total) and protein (animal), corpus-uteri cancer with the same components as breast cancer, prostate cancer with fat (saturated, animal) and protein (animal, total), stomach cancer with fat (fish only) and protein (fish only), lung cancer with cholesterol, and laryngeal cancer with cholesterol. Breast and corpus-uteri cancers also showed significant negative associations with carbohydrate intake. The implications of these findings for future research are discussed.


*Br. J. Cancer* (1981) **44**, 332

### NUTRIENT INTAKES IN RELATION TO CANCER INCIDENCE IN HAWAII

L. N. KOLONEL, J. H. HANKIN, J. LEE, S. Y. CHU, A. M. Y. NOMURA AND M. WARD HINDS

*From the Epidemiology Program, Cancer Center of Hawaii, University of Hawaii, Honolulu, Hawaii* 96813

Received 3 March 1981 Accepted 21 May 1981

**Summary.—A representative sample of 4657 adults ≥45 years of age from the 5 main ethnic groups in Hawaii (Caucasians, Japanese, Chinese, Filipinos and Hawaiians) were interviewed during 1977–1979 regarding their diets. Quantitative food-consumption histories were obtained, from which average daily intakes of fat (saturated, unsaturated, cholesterol, meat, dairy, fish, animal, vegetable and total), protein (animal, meat, fish, dairy and total), carbohydrate, and vitamins A and C (including supplements) were calculated using food-consumption data from standard sources. Multiple regression analysis, with sex as a controlled variable, was used to assess the statistical relationship between these ethnic–sex-specific intakes and corresponding population-based cancer incidence rates of 15 selected sites for which nutrient components are suspected to be either causal or protective. Based on pre-set criteria for establishing important relationships, significant positive associations were found for 6 of the cancer sites: breast cancer with fat (saturated, unsaturated, animal, total) and protein (animal), corpus-uteri cancer with the same components as breast cancer, prostate cancer with fat (saturated, animal) and protein (animal, total), stomach cancer with fat (fish only) and protein (fish only), lung cancer with cholesterol, and laryngeal cancer with cholesterol. Breast and corpus-uteri cancers also showed significant negative associations with carbohydrate intake. The implications of these findings for future research are discussed.**

EPIDEMIOLOGICAL RESEARCH on the relationship of diet to cancer has been limited by the difficulty of adequately assessing exposure. Initial suggestions of dietary associations with cancer have usually resulted from ecological analyses in which international food-disappearance data were correlated with cancer-mortality data (Lea, 1967; Stocks, 1970; Howell, 1974; Armstrong & Doll, 1975). Such indicators of possibly meaningful relationships need to be followed by more refined analyses, since both the exposure data and the outcome data are at best approximate. *Per capita* intakes of foods and nutrients based on food-disappearance data, for example, assume that all foods produced or imported, and not fed to livestock, lost in storage or exported are fully consumed by the population. Such data do not account for home-produced foods and waste in the home; furthermore, they cannot distinguish between the intakes of infants, children, young adults and the elderly, or between men and women. This could result in considerable misrepresentation in a comparison of two countries with very different population structures and food-production practices. The use of international data on morbidity and mortality for such studies also presents problems because of non-comparability

Reprint requests to: Dr L. N. Kolonel, Epidemiology Program, Cancer Center of Hawaii, University of Hawaii, Honolulu, HI 96813.

among countries in completeness of death (or morbidity) registration, diagnostic practices, and medical treatment affecting survival.

Many of these limitations in ecological correlations can be overcome if the dietary information is based on individual interviews rather than national *per capita* consumption estimates, if the comparison groups are located within a single geographic area in which standards of public health and medical practice are uniform, if age-specific analyses can be carried out, and if death or morbidity registration is complete. Hawaii meets all of these requirements. In addition, its multi-ethnic population provides both variations in site-specific cancer risks and considerable diversity in dietary practices. Accordingly, in 1977, we began to collect dietary information on a representative sample of the population of Hawaii for correlation with ethnic-specific cancer incidences from the population-based Hawaii Tumor Registry (HTR).

### METHODS

*Data sources.*—The subjects for this study were part of a random 2% household survey conducted annually by the Hawaii Department of Health (DOH) to collect demographic and health-related data by personal interview. Participation in this survey is very high because it is conducted under statutory provisions. For our study, the DOH interviewers identified all surveyed households on the Island of Oahu (containing 80% of the population of Hawaii) in which male and female adults 45 years or older and belonging to one of the 5 main ethnic groups (Caucasian, Japanese, Hawaiian, Filipino, or Chinese) resided. These households were then contacted by our own interviewers, who arranged for home interviews on diet and other factors.

The dietary interview included information on usual weekly intake of 83 food items which had been selected to cover the main sources of dietary fat and protein (representing 85–90% of total intake), and somewhat less complete information on carbohydrate, vitamin A and vitamin C. Vitamin supplements were also included. The selection of these food items and the determination of typical portion sizes was based on careful review of 4-day measured food records maintained by a group of 100 women representing these ethnic groups. To assist the subjects in estimating food quantities, the interviewers used a series of coloured photographs showing foods in 3 typical portion sizes (small, medium, large) of known weight. The actual plates and bowls used in the pictures were shown to the subjects in order to give a sense of scale. From the photographs, the subjects selected the individual portions or combinations of portions most representative of what they usually ate. The interviewers received extensive training to ensure the collection of complete and unbiased information, and were provided with detailed protocols containing food items to be recorded, equivalent weights and measures, and materials for probing to gain fuller information. As part of the quality-control procedures for the study, they were also accompanied periodically in the field by a supervisor.

The data on diet, as well as demographic information such as age, sex and ethnicity, were coded and keypunched for computer analysis. Individual nutrient intakes were computed from the quantitative consumption information using food composition data from the U.S.D.A. (U.S. Dept of Agriculture, 1972), Japan (Japan Dietetic Assn, 1964), the Philippines (Food and Nutrition Research Center, 1968), Hawaii (Miller & Branthoover, 1957), commercial sources, and home recipes. Ethnic–sex-specific average annual incidences per 100,000 for ages 45 and older during the period 1973–1977 were calculated from incidence data provided by the HTR, and from population data for 1975 provided by the DOH, based on local intercensal estimates.

Fifteen different cancer sites were examined in relation to the nutrient intakes: oesophagus, stomach, colon, rectum, pancreas, larynx, lung, urinary bladder, kidney, thyroid, prostate, breast, corpus uteri, cervix, and ovary. These sites were chosen because of suspected dietary associations, either causal or protective (Lea, 1967; Stocks, 1970; Howell, 1974; Armstrong & Doll, 1975; Carroll & Khor, 1975; Sporn *et al.*, 1976). The 17 nutrient components examined included: fat (total, vegetable, animal, meat, fish, dairy, saturated, unsaturated, cholesterol), protein (total, animal, meat, fish, dairy), carbohydrate and vitamins A and C (including vitamin supplements).

*Statistical analysis.*—The age-specific (45–54, 55–64, 65–74, 75+ years) and age-adjusted mean intake of each nutrient, and the incidence rate of each cancer site were computed for the 10 ethnic–sex-specific groups. Age-adjustment of nutrient intake was done by analysis of covariance; age-adjustment of incidence rates was done by the direct method using the age-distribution of the combined population as the standard.

Preliminary intercorrelation analysis showed that a person's gender was materially related to both nutrient intakes and incidence rates. These results suggested that sex was a strong confounder that could distort apparent relationships of nutrient intakes on incidence rates. Accordingly, to assess the statistical relationship between incidence rate and nutrient intake, we carried out multiple-regression analysis with the ethnic–sex-specific incidence rate as dependent variable, the corresponding mean nutrient intake as independent variable, and sex as a controlled variable. Hence, the indices quantifying the relation of incidence rate to nutrient intake are the partial regression and correlation coefficients statistically adjusted for sex.

We did not use statistical significance as a criterion for judging relationships of interest. Since there were 1275 relationships to consider (15 cancer sites with 17 nutrients, 4 age-specific groups and 1 age-adjusted group), a number of spurious relationships would undoubtedly have turned out to be statistically significant at the conventional 5% probability level. Instead, we chose to establish *a priori* certain criteria for identifying relationships of potential interest. The basis for the selection of these criteria is discussed later. The 3 criteria we applied to the regression lines were: (1) Magnitude of effect; a sex-adjusted increase of 10% in the intake level of the nutrient above the mean value for the entire sample must result in a corresponding change of 20% or more in the incidence rate for cancer in at least 3 of the 4 age-specific groups (45–54 years, 55–64 years, 65–74 years, and $\geq 75$ years); (2) Consistency of association; the sex-adjusted direction (sign) of this relationship must be consistent across all 4 age groups; (3) Strength of association: the sex-adjusted partial correlation coefficient ($r$) must be $\geq 0{\cdot}71$ across all 4 age groups.

### RESULTS

During the period 1977–79, interviews were completed on 4657 subjects. The distribution of the final sample by age, ethnicity and sex is shown in Table I. The overall refusal rate was 13·5%, and did not differ between men and women. By ethnic group, the refusal rates were: Caucasian 17·8%, Japanese 12·6%, Chinese 17·0%, Filipino 5·4%, and Hawaiian 13·4%. It can be seen in Table I that substantial numbers of persons from all ethnic groups were interviewed. The distribution by age and sex corresponds well with that for Oahu as a whole, though the Japanese are over-represented by an estimated 5–10% in the sample. Nevertheless, since the numbers of subjects in all groups are sufficiently large, we are con-

TABLE I.—*Distribution of study group by age, sex and ethnicity*

| Ethnic group | | Age group and sex: 45–54 Male | 45–54 Female | 55–64 Male | 55–64 Female | 65–74 Male | 65–74 Female | 75+ Male | 75+ Female | Total Male | Total Female |
|---|---|---|---|---|---|---|---|---|---|---|---|
| Caucasian | No. | 250 | 226 | 203 | 183 | 114 | 89 | 37 | 45 | 604 | 543 |
| | (%) | (27·7) | (22·2) | (27·4) | (25·0) | (25·4) | (23·4) | (18·5) | (19·3) | (26·3) | (23·0) |
| Japanese | No. | 356 | 474 | 337 | 353 | 136 | 153 | 93 | 122 | 922 | 1102 |
| | (%) | (39·4) | (46·6) | (45·5) | (48·2) | (30·3) | (40·2) | (46·5) | (52·4) | (40·2) | (46·6) |
| Chinese | No. | 69 | 57 | 54 | 64 | 52 | 43 | 25 | 34 | 200 | 198 |
| | (%) | (7·6) | (5·6) | (7·3) | (8·7) | (11·6) | (11·3) | (12·5) | (14·6) | (8·7) | (8·4) |
| Filipino | No. | 130 | 125 | 76 | 59 | 113 | 38 | 39 | 19 | 358 | 241 |
| | (%) | (14·4) | (12·3) | (10·3) | (8·1) | (25·2) | (10·0) | (19·5) | (8·2) | (15·6) | (10·2) |
| Hawaiian | No. | 99 | 136 | 70 | 73 | 34 | 58 | 6 | 13 | 209 | 280 |
| | (%) | (11·0) | (13·4) | (9·5) | (10·0) | (7·6) | (15·2) | (3·0) | (5·6) | (9·1) | (11·8) |
| Total | No. | 904 | 1018 | 740 | 732 | 449 | 381 | 200 | 233 | 2293 | 2364 |
| | (%) | (100) | (100) | (100) | (100) | (100) | (100) | (100) | (100) | (100) | (100) |

TABLE II.—*Significant associations of age-adjusted mean daily nutrient intakes and average annual cancer incidence (per 100,000) for 10 ethnic-sex groups in Hawaii, based on multiple regression analysis and selected criteria (see text)*

| Cancer site | Fat† Component | b* | r‡ | Protein† Component | b | r | Complex carbohydrate† b | r |
|---|---|---|---|---|---|---|---|---|
| Lung | Cholesterol | 0·30 | 0·94 | | | | | |
| Larynx | Cholesterol | 0·20 | 0·76 | | | | | |
| Breast | Total | 1·49 | 0·94 | | | | | |
| | Animal | 2·50 | 0·89 | Animal | 2·34 | 0·92 | −0·37 | −0·71 |
| | Saturated | 4·47 | 0·95 | | | | | |
| | Unsaturated | 2·59 | 0·90 | | | | | |
| Corpus uteri | Total | 0·60 | 0·98 | | | | | |
| | Animal | 1·06 | 0·98 | Animal | 0·94 | 0·96 | −0·16 | −0·82 |
| | Saturated | 1·79 | 1·00 | | | | | |
| | Unsaturated | 1·05 | 0·95 | | | | | |
| Prostate | | | | Total | 1·06 | 0·78 | | |
| | Animal | 0·87 | 0·90 | Animal | 0·76 | 0·83 | | |
| | Saturated | 1·27 | 0·87 | | | | | |
| Stomach§ | Fish | 10·21 | 0·92 | Fish | 3·18 | 0·73 | | |

† Units for nutrient intakes as follows: fat (except cholesterol), protein and carbohydrate in g/day; cholesterol in mg/day.
* Partial regression coefficient, adjusted for sex.
‡ Partial correlation coefficient, adjusted for sex.
§ Significant associations only after elimination of first criterion (see text).

fident that the data on nutrient intakes are representative.

In accordance with the 3 selection criteria defined above, 9 of the 15 cancer sites (oesophagus, colon, rectum, pancreas, kidney, bladder, ovary, cervix and thyroid) showed no potentially important associations with any of the nutrients examined. The remaining 6 cancer sites showed important associations with only selected components of the 3 major nutrients. For those associations which met the 3 selection criteria, the sex-partial regression and correlation coefficients of age-adjusted cancer rates or age-adjusted mean nutrient intakes are presented in Table II. At least one component of fat was associated positively with all 6 of the cancers. The association for lung and laryngeal cancers was seen only with cholesterol and for stomach cancer only with fish fat, whereas for the 3 sex-related cancers the association was seen for several different components of fat. Protein intake was associated positively with the same 3 sex-related cancers. Complex carbohydrate showed negative associations with breast and corpus-uteri cancers. Vitamins A and C did not show potentially important associations with any of the 15 cancer sites.

Because of the possibility that some of these apparent associations might have resulted from one or more outlying values, we examined the scattergrams for the various relationships in Table II. In no instance, however, could outliers account for the relationship. Selected scattergrams for a few of these relationships are shown in the figure.

Table III shows the sex-adjusted intercorrelations among the main nutrient categories. There were high positive partial correlation coefficients for protein with fat, fat with both vitamins, and vitamin A with C. Notable negative correlations included fat with carbohydrate, and carbohydrate with vitamin A.

### DISCUSSION

Although this study has certain advantages over the usual geographic analyses (as noted earlier), it is still subject to the well-known "ecological correlation fallacy"

FIGURE.—Age-adjuested incidence rates of cancer in relation to selected nutrient intakes among 5 ethnic groups in Hawaii. △, Males; ○, females.

TABLE III.—*Sex-adjusted correlation matrix (partial* r) *for selected major nutrients*

| | Total fat | Carbo-hydrate | Total vitamin A | Total vitamin C |
|---|---|---|---|---|
| Total protein | 0·91 | −0·55 | 0·74 | 0·75 |
| Total fat | — | −0·82 | 0·80 | 0·77 |
| Carbohydrate | | — | −0·72 | −0·62 |
| Total vitamin A | | | — | 0·97 |

(Robinson, 1950). Also, it is reasonable to assume that any specific nutrient–cancer relationship is potentially confounded by sex, age, ethnicity, intake of other nutrients, and other sociodemographic characteristics. Whereas we statistically controlled for age and sex, the constraints of our data precluded our controlling for other potential confounders.

Our use of multiple regression and correlation analysis was based on the belief that even with 10 data points (occasionally 5 for sex-specific sites) we could nevertheless make observations of heuristic value, since our purpose was primarily to confirm results from other, weaker ecological correlation analyses, and to identify possible new relationships for further diet-related cancer research. It is worth noting that the interviewed sample was large and that the incidence rates were population-based, covering the entire state; thus, data points used in the analysis were probably relatively stable.

The decision to use pre-set criteria to establish meaningful relationships was in part a practical one. As noted above, with 1275 separate regression equations to consider, simply testing the regression coefficients for statistical significance at the 5% level would have yielded a substantial number of "significant" relationships by chance alone (Type I error). We preferred to improve the likelihood of our focusing on the truly important relationships by applying some reasoned judgment to the data. First, we decided that relationships would not be of practical significance if relatively large changes in the intake of the nutrient had little impact on the incidence of the associated cancer. Since neither the magnitude of the incidence rates for the different sites nor the range of variation among the different ethnic groups was uniform, a single meaningful value of the partial regression coefficient "b" could not be chosen for all relationships (see Criterion I under Statistical Analysis in Methods). We did allow for 1 of the 4 age-specific groups to fail this criterion, in order to avoid our overlooking certain potentially meaningful relationships by an excessive strictness in these pre-set requirements. Secondly, we decided that consistency in the association across the 4 age groups was important, since all groups were adults and could be expected to show similar relationships (see Criterion 2). Finally, we decided that a minimal value for the partial correlation coefficient of the regression equation would help establish the potential importance of a relationship. We chose the partial $r$ value of $\geq 0{\cdot}71$, since it indicates that at least 50% of the variation in the incidence can be accounted for by the related nutrient (see Criterion 3). Because most correlation studies rely only on the $b$ and/or $r$ values to assess the significance of relationships, we also examined our data with the omission of Criterion I (magnitude of effect). Interestingly, this identified only one additional relationship, namely, the association of fish nutrient sources with stomach cancer.

Although all 15 sites examined were selected because of reported or suspected relationships to diet, only 6 showed notable associations in the present analysis. Furthermore, these associations were only with a few selected nutrient factors in each case. This degree of specificity in our analysis suggests that the associations we did detect are worth further consideration. The lack of association with certain nutrients examined, such as vitamins A and C, is also notable. There are two possible explanations for this particular negative finding. First, our dietary schedule was not as complete on food sources for these two vitamins as it was for fat and protein. Second, since these vitamins are considered to be anticarcinogens (Mirvish *et al.*, 1972; Sporn *et al.*, 1976) their effects on cancer incidence depend on exposure to carcinogenic agents. Thus, for example, a possible negative association of vitamin C with stomach cancer might best be seen only after controlling for nitrosamine precursors in the diet.

Lung and laryngeal cancers showed similar positive associations with cholesterol. A positive correlation of lung-cancer mortality in 40 countries with *per capita* daily fat intake in males has been reported (Carroll & Khor, 1975). Since both lung and laryngeal cancers are strongly associated with smoking (Hammond, 1966) it is possible that the association with cholesterol is indirect, reflecting some underlying pattern of eating common to many smokers. Laryngeal cancer has been associated with alcohol consumption, which, in turn, is highly correlated with cigarette smoking (Hinds *et al.*, 1980). Thus, an adverse effect of ethanol on cholesterol metabolism leading to cancer might be considered. Studies in animals have shown that ethanol alters the metabolism of cholesterol in tissues (Rothfeld *et al.*, 1975).

The only gastrointestinal site which showed any important nutrient associations was stomach cancer. Notably, colon and rectal cancers did not appear. Although colon cancer has been associated positively with dietary fat and/or meat consumption in international geographic correlations and in case-control studies (Haenszel *et al.*, 1973; Armstrong & Doll, 1975; Howell, 1975; Jain *et al.*, 1980) other epidemiological studies have reported negative results for these same dietary factors (Wynder & Shigematsu, 1967; Modan *et al.*, 1975; Haenszel *et al.*, 1980). The primary reason for the lack of an association in our analysis is that the Hawaiians have very low rates of colon and rectal cancers yet consume very high-fat diets, while the Japanese have high rates of these cancers but relatively low fat intakes.

It is interesting that the only important relationship for stomach cancer was with fat and protein intake specifically from fish sources, and that stomach was the only site which showed this particular association. Other studies have shown an association of stomach cancer with the consumption of dried/salted fish (Haenszel *et al.*, 1972; Bjelke, 1974), and a carcinogenic mechanism related to the formation of nitrosamines from the high content of secondary amines and nitrate in these products has been proposed (Correa *et al.*, 1975; Lijinsky, 1977). Our analysis points to the same food source, and is certainly consistent with the nitrosamine hypothesis.

Three sex-related cancers showed associations in the analysis: breasts, corpus uteri, and prostate. Positive associations of these cancers with fat and protein intake have been reported in other studies (Drasar & Irving, 1973; Howell, 1974; Armstrong & Doll, 1975). Whereas breast and corpus-uteri cancers showed positive associations with both saturated and unsaturated fats, prostate cancer showed the association for saturated fats only, suggesting greater specificity. Biological mechanisms for the association of fat intake or obesity with breast and endometrial cancers have been proposed, including the conversion in fat tissue of endogenously produced androstenedione to oestrone, a potentially carcinogenic oestrogen (Schindler *et al.*, 1972; MacDonald *et al.*, 1978), and the effect of fat intake in increasing secretion of prolactin, a possible carcinogen for breast tissue (Hill & Wynder, 1976).

Although our analysis was based on independent associations of nutrients with cancer incidence, there are significant intercorrelations among these nutrients (Table III). Thus, fat and protein were highly correlated ($r = 0{\cdot}91$), which could explain why the sites associated with fat were generally also associated with protein. (On the other hand, an independent effect of protein cannot be dismissed, since animal studies have shown, for example, that diets reduced in protein but controlled for caloric intake to maintain body weight yield fewer mammary tumours in mice (Tannenbaum & Silverstone, 1953).) Similarly, the negative correlation between carbohydrate and fat intakes ($r = 0{\cdot}82$) and the positive association of fat with breast and corpus-uteri cancers could readily explain the negative association of these sites with carbohydrate.

Intercorrelations among cancer sites may provide useful clues to common aetiologies (Winkelstein *et al.*, 1977). Examination of our data, however, did not reveal any unusual correlations among sites. In fact, the well known correlation between breast and colon cancers (Howell, 1976) was not seen, primarily because Hawaiian women have very high breast-cancer rates but very low colon-cancer rates. This discrepancy suggests that the speculation of an aetiological association of dietary fat with both these cancers may be incorrect.

Based on these analyses, a number of additional studies, particularly case-control analyses, would appear worth pursuing. Certainly, the associations between fat and the 3 sex-related cancers (breast, corpus uteri, prostate) should be studied further, and the possible role of nutrients in smoking-related cancers is of interest (especially with continued high rates of smoking and the consequent need to understand more about cancer risks among smokers). As a result of preliminary analyses on the data reported here, we have already begun a number of projects in Hawaii, including case-control studies of dietary factors in relation to cancers of the breast, prostate, bladder and lung. We are also continuing to follow the entire cohort of $\sim 5{,}000$ interviewed subjects for future cancer occurrence. This will enable us to assess cancer risk directly on the basis of antecedent dietary information and to control for a number of confounding factors, such as ethnicity and smoking. Furthermore, it will provide us with an unusual opportunity to compare the results of aggregate correlational analyses with individual follow-up information based on the same data sets. Such a comparison will provide a direct test of the usefulness of ecological correlations in epidemiological research on diet and cancer.

This work was supported in part by Grants No. 1 RO1 CA 20897 and 1 NO1 CA 15655 and Contract No. NO1-CP-53511 from the National Cancer Institute, National Institutes of Health.

We would like to acknowledge the interviewing staff of the Epidemiology Program, Cancer Center of Hawaii, for collection of the data, and Mrs C. Brotherton, Mrs M. Ng, and the staff of the Health Surveillance Program, Hawaii Department of Health, for technical assistance.

### REFERENCES

ARMSTRONG, B. & DOLL, R. (1975) Environmental factors and cancer incidence and mortality in different countries, with special reference to dietary practices. *Int. J. Cancer*, **15**, 617.

BJELKE, E. (1974) Epidemiologic studies of cancer of the stomach, colon, and rectum with special emphasis on the role of diet. *Scand. J. Gastroenterol.*, **9** (Suppl. 31), 1.

CARROLL, K. K. & KHOR, H. T. (1975) Dietary fat in relation to tumorigenesis. *Progr. Biochem. Pharmacol.*, **10**, 308.

CORREA, P., HAENSZEL, W., CUELLO, C., TANNENBAUM, S. & ARCHER, M. (1975) A model for gastric cancer epidemiology. *Lancet*, ii, 58.

DRASAR, B. S. & IRVING, D. (1973) Environmental factors and cancer of the colon and breast. *Br. J. Cancer*, **27**, 167.

FOOD AND NUTRITION RESEARCH CENTER (1968) *Food Composition Table for Use in the Philippines, 4th Revision*. Manila: National Science Development Board.

HAENSZEL, W., KURIHARA, M., SEGI, M. & LEE, R. K. C. (1972) Stomach cancer among Japanese in Hawaii. *J. Natl Cancer Inst.*, **49**, 969.

HAENSZEL, W., BERG, J. W., SEGI, M., KURIHARA, M. & LOCKE, F. B. (1973) Large bowel cancer in Hawaiian Japanese. *J. Natl Cancer Inst.*, **51**, 1765.

HAENSZEL, W., LOCKE, F. B. & SEGI, M. (1980) A case-control study of large bowel cancer in Japan. *J. Natl Cancer Inst.*, **64**, 17.

HAMMOND, E. C. (1966) Smoking in relation to the death rates of 1 million men and women. In *Epidemiological Approaches to the Study of Cancer and Other Chronic Diseases. Natl Cancer Inst. Monogr.*, **19**, 127.

HILL, P. & WYNDER, E. (1976) Diet and prolactin release. *Lancet*, ii, 806.

HINDS, M. W., KOLONEL, L. N., LEE, J. & HIROHATA, T. (1980) Associations between cancer incidence and alcohol/cigarette consumption among five ethnic groups in Hawaii. *Br. J. Cancer*, **41**, 929.

HOWELL, M. A. (1974) Factor analyses of international cancer mortality data and *per capita* food consumption. *Br. J. Cancer*, **29**, 328.

HOWELL, M. A. (1975) Diet as an etiological factor in the development of cancers of the colon and rectum. *J. Chron. Dis.*, **28**, 67.

HOWELL, M. A. (1976) The association between colorectal cancer and breast cancer. *J. Chron. Dis.*, **29**, 243.

JAIN, M., COOK, G. M., DAVIS, F. G., GRACE, M. G., HOWE, G. R. & MILLER, A. B. (1980) A case-control study of diet and colorectal cancer. *Int. J. Cancer*, **26**, 757.

JAPAN DIETETIC ASSOCIATION (1964) *Standard Tables of Food Composition*. Tokyo: Daiichi Shuppon Co.

LEA, A. J. (1967) Neoplasms and environmental factors. *Ann. R. Coll. Surg. Engl.*, **41**, 432.

LIJINSKY, W. (1977) Nitrosamines and nitrosamides in the etiology of gastrointestinal cancer. *Cancer*, **40**, 2446.

MACDONALD, P. C., EDMAN, C. D., HEMSELL, D. L., PORTER, J. C. & SIITERI, P. K. (1978) Effect of obesity on conversion of plasma androstenedione to estrone in postmenopausal women with and without endometrial cancer. *Am. J. Obstet. Gynecol.*, **130**, 448.

MILLER, C. D. & BRANTHOOVER, B. (1957) Nutritive values of some Hawaii foods. Honolulu: Hawaii Agric. Expt Station No. 52.

MIRVISH, S. S., WALLCAVE, L., EAGEN, M. & SHUBIK, P. (1972) Ascorbate–nitrite reaction: Possible means of blocking the formation of carcinogenic N-nitroso compounds. *Science*, **177**, 65.

MODAN, B., BARRELL, V., LUBIN, F., MODAN, M., GREENBERG, R. A. & SAXON, G. (1975) Low-fiber intake as an etiologic factor in cancer of the colon. *J. Natl Cancer Inst.*, **55**, 15.

ROBINSON, W. S. (1950) Ecological correlations and the behavior of individuals. *Am. Soc. Rev.*, **15**, 351.

ROTHFELD, B., VARADY, A., JR, MARGOLIS, S. & 4 others (1975) The effect of ethanol and high cholesterol diet on tissue lipids. *Biochem. Med.*, **13**, 276

SCHINDLER, A. E., EBERT, A. & FRIEDRICH, E. (1972) Conversion of androstenedione to estrone by human fat tissue. *J. Clin. Endocrinol. Metab.*, **35**, 627.

SPORN, M. B., DUNLOP, N. M., NEWTON, D. L. & SMITH, J. M. (1976) Prevention of chemical carcinogeneis by vitamin A and its synthetic analogs (retinoids). *Fed. Proc.*, **35**, 1332.

STOCKS, P. (1970) Cancer mortality in relation to national consumption of cigarettes, solid fuel, tea and coffee. *Br. J. Cancer*, **24**, 215.

TANNENBAUM, A. & SILVERSTONE, H. (1953) Mammary carcinoma in the mouse. *Proc. Am. Assoc. Cancer Res.*, **1**, 56.

U.S. DEPT OF AGRICULTURE, AGRICULTURAL RESEARCH SERVICE (1972) Data Set 8-1-1, *Composition of Foods, Raw, Processed, Prepared*. Washington, D.C.: U.S.D.A.

WINKELSTEIN, W., SACKS, S. T., ERNSTER, V. L. & SELVIN, S. (1977) Correlations of incidence rates for selected cancers in the nine areas of the Third National Cancer Survey. *Am. J. Epidemiol.*, **105**, 407.

WYNDER, E. L. & SHIGEMATSU, T. (1967) Environmental factors of cancer of the colon and rectum. *Cancer*, **20**, 1520.